# Congenital Long QT Syndrome: An Update and Present Perspective in Saudi Arabia

**DOI:** 10.3389/fped.2013.00039

**Published:** 2013-11-20

**Authors:** Zahurul A. Bhuiyan, Safar Al-Shahrani, Jumana Al-Aama, Arthur A. M. Wilde, Tarek S. Momenah

**Affiliations:** ^1^Laboratoire de Génétique Moléculaire, Service de Génétique Médicale, Centre Hospitalier Universitaire Vaudois, Lausanne, Switzerland; ^2^Department of Pediatrics, Faculty of Medicine, King Khalid University, Abha, Saudi Arabia; ^3^Princess Al Jawhara Albrahim Center of Excellence in Research of Hereditary Disorders, Jeddah, Saudi Arabia; ^4^Department of Genetic Medicine, King Abdulaziz University, Jeddah, Saudi Arabia; ^5^Department of Cardiology, Academic Medical Center, University of Amsterdam, Amsterdam, Netherlands; ^6^Department of Pediatric Cardiology, King Fahad Medical City, Riyadh, Saudi Arabia

**Keywords:** arrhythmia, long QT syndrome, genetics, consanguinity

## Abstract

Primary cardiac arrhythmias are often caused by defects, predominantly in the genes responsible for generation of cardiac electrical potential, i.e., cardiac rhythm generation. Due to the variability in underlying genetic defects, type, and location of the mutations and putative modifiers, clinical phenotypes could be moderate to severe, even absent in many individuals. Clinical presentation and severity could be quite variable, syncope, or sudden cardiac death could also be the first and the only manifestation in a patient who had previously no symptoms at all. Despite usual familial occurrence of such cardiac arrhythmias, disease causal genetic defects could also be *de novo* in significant number of patients. Long QT syndrome (LQTS) is the most eloquently investigated primary cardiac rhythm disorder. A genetic defect can be identified in ∼70% of definitive LQTS patients, followed by Catecholaminergic Polymorphic Ventricular Tachycardia (CPVT) and Brugada syndrome (BrS), where a genetic defect is found in <40% cases. In addition to these widely investigated hereditary arrhythmia syndromes, there remain many other relatively less common arrhythmia syndromes, where researchers also have unraveled the genetic etiology, e.g., short QT syndrome (SQTS), sick sinus syndrome (SSS), cardiac conduction defect (CCD), idiopathic ventricular fibrillation (IVF), early repolarization syndrome (ERS). There exist also various other ill-defined primary cardiac rhythm disorders with strong genetic and familial predisposition. In the present review we will focus on the genetic basis of LQTS and its clinical management. We will also discuss the presently available genetic insight in this context from Saudi Arabia.

## Introduction

Familial or hereditary cardiac arrhythmias comprise significant percentages of arrhythmias and also causal to sudden cardiac death (SCD) (1, 2). During the last two decades, scientists and clinicians have provided enormous effort to unravel the intricate and complex mechanisms of congenital familial arrhythmias (1–8). To understand the mechanism of arrhythmogenesis, we need to know the basics of cardiac cellular structure and their electrophysiological properties. Cardiac myocytes are the major functioning cells in the heart and they are extensively coupled so that impulses propagate rapidly and uniformly. Cardiomyocytes are separated from each other by a specialized boundary called the intercalated disk; gap junction proteins, cardiac desmosomes, and ion channels are located in the intercalated disk (9). Gap junctions consist of tightly packed connexins which permit intercellular exchange of small molecules and also permit to flow the excitatory currents from one cell to its neighboring cell. Desmosomes along with the adherens junctions are responsible for the mechanical attachments of individual cardiomyocytes. All these components in the intercalated disks are sequentially segregated and each component exerts its unique function; disruption of the one component affects the function of other components, which predisposes the heart to develop arrhythmias (9–11). Cardiac ion channels are pore-forming protein complexes that provide voltage gated and intricately coordinated inward and outward movement of ionic currents across the cell membranes, essential for cardiac rhythm generation and propagation. Long QT syndrome (LQTS), short QT syndrome (SQTS), sick sinus syndrome (SSS), cardiac conduction defect (CCD), BrS, catecholaminergic polymorphic ventricular tachycardia (CPVT), early repolarization syndrome (ERS), and familial Atrial Fibrillation (AF) are the presently known cardiac channelopathies, which could occur due to a single or multiple defect in the genes linked to cardiac rhythm generation and propagation.

In this review, we will describe exclusively on LQTS linked arrhythmias, its pathophysiology and presently available clinical management. We have pioneered in elucidating genetic pathology in familial cardiac arrhythmias in Saudi Arabia (1, 12–14), we are still conducting cardiogenetic investigations in Saudi Arabia, at the end of this review, we will discuss the presently available knowledge about the genetic and clinical findings obtained from several Saudi Arabian families with history of syncope and SCD. We will also add the recently published data on LQTS from a team at King Faisal Specialist Hospital and Research Centre, Riyadh (15).

## Long QT Syndrome

Congenital LQTS is an inherited disorder defined by prolongation of the QT interval on electrocardiogram (ECG). Patients with all forms of LQTS are predisposed to the ventricular tachyarrhythmia, torsades de pointes (TdP) leading to recurrent syncope, or SCD. In many instances, syncope or sudden death could be the first and the only manifestation. LQTS affects an estimated 1 in 2,000 people worldwide (16). The hallmark of the LQTS is prolongation of the QT interval on ECG (corrected for heart rate, i.e., QTc). Normal values of QTc are 440 ms in males and 450 ms in females. In children, age and gender dependent values are relevant. Recent consensus recommendation for LQTS diagnosis are the following (17, 18):

1. LQTS is diagnosed:
  - In the presence of an LQTS risk score ≥3.5 in the absence of a secondary cause for QT prolongation and/or
  - In the presence of an unequivocally pathogenic mutation in one of the LQTS genes or
  - In the presence of a QT interval corrected for heart rate using Bazett’s formula (QTc) ≥500 ms in repeated 12-lead electrocardiogram and in the absence of a secondary cause for QT prolongation.
2. LQTS can be diagnosed in the presence of a QTc between 480 and 499 ms in repeated 12-lead ECGs in a patient with unexplained syncope in the absence of a secondary cause for QT prolongation and in the absence of a pathogenic mutation.

Patients with QTc duration ≥500 ms had a significantly higher cumulative probability of experiencing their first syncope as compared to patients with QTc duration <500 ms from birth through age 20 years (19). But, patients who have experienced 2, 3, and 4 syncope episodes, the risk of a subsequent syncope episode was virtually identical between patients who had narrow or prolonged QTc duration (19). The molecular basis of LQTS is heterogeneous and to date, mutations in 13 different genes have been described causal to LQTS (1–8, 19, 20). A genetic defect is usually found in 70% of the LQTS patients in one of these 13 genes (1–8, 19, 20). Among the presently known 13 different types of LQTS, the most common are LQTS1, LQTS2, and LQTS3, due to defects in cardiac ion channel genes, *KCNQ1, KCNH2*, and *SCN5A*, respectively. Ninety percent of all the LQTS causal mutations are found in these three genes (1–8, 19, 20). Mutations in the remaining 10 genes are rare and comprise only ∼10% of all the presently known LQTS mutations.

Long QT syndrome is usually an autosomal dominant disease, but, occasionally, multiple mutations in a single gene or in different genes could be found in 5–10% of the patients with LQTS (21, 22). Patients with multiple mutations could exhibit a longer QTc compared with those with a single mutation and such patients are also at ∼3.5-fold increased risk for life-threatening cardiac events (21, 22)

### LQTS1

**Figure 1:**
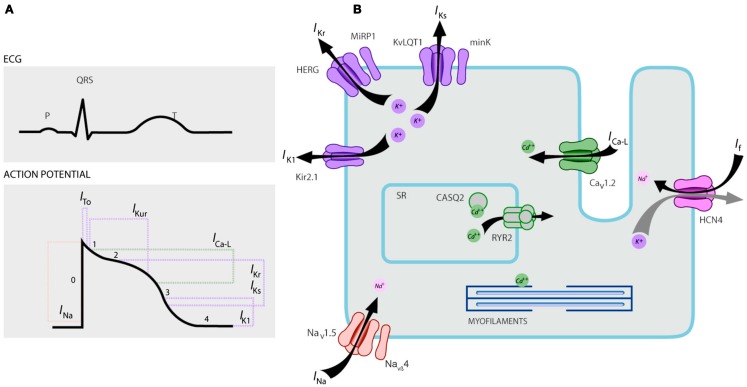
**Ionic currents contributing to the ventricular action potential (A) and schematic representation of a cardiomyocyte displaying (only) those proteins involved in the pathogenesis of inherited arrhythmia syndromes (B)**. In **(A)**, the action potential is aligned with its approximate time of action during the ECG. In **(B)**, ankyrin-B, an adapter protein involved in the long QT syndrome type 4, is not depicted.

Mutations in the *KCNQ1* gene are the commonest form of all LQTS and referred as LQTS type 1 (LQTS1). Fifty percent of all the LQTS causal mutations are found in the *KCNQ1* gene (20). KvLQT1 (also called Kv7.1) is a protein made by the *KCNQ1* gene and the protein forms tetramers in the endoplasmic reticulum inside the cells, which putatively co-assembles with the protein minK (encoded by *KCNE1*) and are then transported to the plasma membrane of the cardiac myocytes, where they mediate a slowly activating current which accelerates the repolarization of action potential in cardiac tissues, this current is known as IKs (Figure 1). LQTS1 causal *KCNQ1* mutations are mostly missense mutations and in rare instances they could be frameshift mutations in the C-terminal region (23). Cardiac arrhythmia in *KCNQ1* mutation carriers are triggered by adrenergic drives, e.g., emotional stress, physical exertion, diving, swimming (24–26). Patients with mutations at the transmembrane domains of KvLQT1 are at higher risk of LQTS-related cardiac events and have greater sensitivity to sympathetic stimulation (26, 27). Patients with missense mutations are at increased risk compared to patients with non-sense or truncating mutations (27, 28). Variation in the 3′-UTR in the KCNQ1 gene also affects the arrhythmia susceptibility significantly, presumably by impacting on the expression of the gene (29). Patients with arrhythmias due to *KCNQ1* mutations respond quite well to β-blockers, but, some patients could still be less responsive or even resistant to this medication. In a recent article, Barsheshet et al. (30) claimed that the patients with mutations outside the cytoplasmic loop (c-loop) region in the KvLQT1 are less responsive to β-blockers.

Homozygous or compound heterozygous mutations in the *KCNQ1* gene, which could cause the recessive form of the disease, Jervell and Lange-Nielsen syndrome (JLNS), type 1 (31), are quite rare. JLNS patients suffer from severe cardiac arrhythmias and deafness as well (31, 32). Patients with *KCNQ1* mutations causal to JLNS usually do not have any functional IKs (28, 31–33). It is possible that some patients could have no deafness despite having homozygous or compound heterozygous mutations in *KCNQ1*, such cases are referred as autosomal recessive LQTS1 (13, 32). In these patients, small amount of functional IKs current (<10% of total IKs) could still be present, which maintains the hearing function, but, their cardiac rhythm defects are equally severe like in JLNS patients (13, 32).

### LQTS2

This type of LQTS is equally prevalent as LQTS1, accounting for 35–40% of LQTS patients with a detectable mutation (1, 20, 24, 25). *KCNH2* encodes for the HERG protein (Kv11.1), which is the α-subunit of the rapidly activating delayed rectifier K^+^ current (IKr). Pathogenic mutations in this gene that reduces the Kv11.1 channel function prolongs duration of the QT interval (Figure 1), and are causal to LQTS2. Twenty-nine percent of the syncopal attacks in LQTS2 occur during rest/sleep and only 13% of the syncopal attacks were reported to occur during exercise (25, 34). Sudden startling noises, e.g., alarm clock rings, doorbells, telephone rings, typically trigger syncopal attacks in these patients (24, 34). Patients with mutations in the pore-forming region of the Kv11.1 (encoded by *KCNH2* gene) are susceptible to high risk for arrhythmia-related cardiac events compared with patients with non-pore region mutations (35). In neonates, 2:1 Atrioventricular (AV) block is preferentially associated with *KCNH2* mutations (36). Complete AV block complicated by LQTS were also found in 17% of adult patients with a mutation in the *KCNH2* gene (37). Homozygous mutations in *KCNH2* are rare and when present, patients suffer from a severe form of LQTS, with 2:1 AV block and severe ventricular arrhythmias, during intrauterine stages as well as after birth (11, 38–40). In addition to the numerous mutations described in LQTS2 pathology, common polymorphic variants in the *KCNH2* gene could also modulate the disease severity. An intriguing example is K897T polymorphism (SNP) in *KCNH2*, which is present in 33% of the general population (41). K897T was reported to exacerbate the pathogenicity of the *KCNH2* mutations (42, 43).

### LQTS3

Nav1.5 is the pore-forming α-subunit of the voltage-dependent cardiac Na^+^ channel, it is an integral membrane protein encoded by the *SCN5A* gene and is involved in the initiation and conduction of cardiac action potentials. Cardiac Na^+^ channels are composed of a pore-forming α-subunit (encoded by *SCN5A*) and one or more auxiliary β-subunits.

LQTS3 is caused by gain of function mutations that disrupt fast inactivation of the α-subunit (Figure 1) and a mutation in the SCN5A gene have been described in <10% of all LQTS patients with a mutation (1, 20, 24). LQTS3 patients experience the majority (39%) of their cardiac events during sleep/rest (25), and about ∼13% of the events were reported to occur during exercise (25). In several instances, a single *SCN5A* mutation was shown to exert two or even three distinct phenotypes of arrhythmias in the same family, e.g., LQTS, BrS, or CCD (44–47). Male patients with a LQTS3 mutation could develop symptoms much earlier than the female patients (48). Both heterozygous and homozygous *SCN5A* mutations have been described in LQTS3 with functional 2:1 AV block (49). We have recently found an Iranian family with the 1507_1509delQKP mutation in multiple family members, where patients had combined LQTS and CCD (data not shown). This mutation has been reported in patients from other countries as well, which suggests that this is a recurrent and hot spot mutation [(49, 50) and our unpublished data]. Mutations with such a loss- and gain-of-function characteristics during different phases of the action potential were also reported in 1493delK and 1795insD mutations (44, 51).

Occasionally, an SNP could also exert pathogenic effect on its carriers. S1103Y is a common variant in the *SCN5A* gene, present in 13% of the African Americans (52). Carriers with this variant are at increased risk of arrhythmias and sudden infant death syndrome (SIDS) (52).

### LQTS4

LQTS4 represents the first non-channel form of LQTS. A mutation in the ANK2, an adapter protein, leads to intracellular calcium overload which contributes to the LQTS4 (53, 54). In addition to QT prolongation, patients with this syndrome could have sinus bradycardia, paroxysmal AF, and CPVT (54). Pathogenic effect of the *ANK2* mutations could be moderate to severe and the clinical expressions depend on the severity of the mutation.

### LQTS5

Mutations in *KCNE1* are associated with the LQTS5 (Figure 1) (55, 56). Heterozygous *KCNE1* mutations reduce IKs by exerting a dominant negative effect on its accompanying normal allele, and lead to delayed cardiac repolarization (Figure 1), responsible for increased risk of arrhythmias (6). Patients with homozygous *KCNE*1 mutations suffer from JLNS (type 2) (57, 58).

D85N is a polymorphism in the KCNE1 gene, present in 0.7–1% of the general population (41). In a study by Nishio et al. (59), D85N polymorphism was more frequently found in the LQTS patients, making it a risk genotype in LQTS pathology (potentially only in the Asian population). In Europe, D85N was reported in 5% of acquired LQTS (aLQTS) patients (in a cohort of 32 patients), who had experienced TdP (60).

### LQTS6

*KCNE2* gene encodes for MinK-related peptide 1 (MiRP1), a putative β-subunit of the cardiac potassium channel IKr (Figure 1). Mutations in *KCNE2* gene could also lead to defects in the rapidly activating component of the delayed rectifier potassium current (IKr), pathologic basis of LQTS6 (61). Auditory/acoustic stimulus like alarm clock noise, doorbell etc. could provoke syncopal attacks in KCNE2 mutation carriers, similar to *KCNH2* mutation (62).

### LQTS7

This syndrome is also known as Andersen–Tawil syndrome (ATS). ATS is a rare disorder, manifested by occasional syncope, and cardiac arrest. ECG features include mild QT interval prolongation, abnormal *U* waves, frequent ventricular ectopy, bidirectional ventricular tachycardia (VT), and polymorphic VT. This syndrome also exhibits extracardiac features, e.g., skeletal muscle periodic paralysis and developmental problems, such as cleft palate, low set ears, short stature, and developmental defects in the limbs (63). Majority of clinically diagnosed ATS patients were reported to have a mutation in *KCNJ2* (63). *KCNJ2* encodes a pore-forming subunit of inwardly rectifying potassium channels (IK1) (Figure 1) (64, 65).

### LQTS8

Also known as Timothy syndrome (TS), patients show severe QT prolongation on their ECGs, which is combined with syndactyly, baldness at birth, and small teeth in 100% of cases and less penetrant cardiac structural malformations, mental retardation, autism, and facial dysmorphic features (66). There are two subtypes: TS1 (classical) and TS2 (rare form).

TS2 is cardiologically severer than TS1 (66, 67). TS2 patients also lack syndactyly (67). Mutations in the α-1 subunit of the L-type calcium current (ICa-L) encoding gene CACNA1C lead to both forms of TS (LQTS8). There are alternatively spliced, mutually exclusive exon-8 in the *CACNA1C* gene, for clarity, they are named as exon-8 and exon-8A. *In* heart and brain, where the *CACNA1C* is predominantly expressed, exon-8 is found in ∼80% of the mRNA transcripts and exon-8A is present ∼20% transcripts (66). G406R mutation in exon-8A is causal to classic form of TS (TS1) and G402S in exon-8 was reported in severer in TS2. A new addition to this list is Ala1473Gly mutation, which has been described in a TS infant with further expanded phenotype (68). All mutations were either *de novo* or mosaic in the parent, and all result gain of function of the ICa-L channel (66–69).

### LQTS9 and LQTS10

Mutations in *CAV3* or *SCN4B* produce gain of function in late INa, causing an LQTS3-like phenotype (70–74). They are known as LQTS9 (associated with CAV3 mutation) and LQTS10 (associated with *SCN4B* mutation).

Caveolae are nicely described by Engelman et al. (72) as “little caves” in the plasma membrane. They are small uncoated pits and is considered as the site of important dynamic and regulatory events at the plasma membrane (72, 73). Caveolins are the principal proteins in the caveolae, and caveolin-3 (encoded by gene *CAV3*) is specifically found in cardiomyocytes and skeletal muscle cells. Several cardiac ion channels have been specifically reported to be localized in the caveolae extracted from cardiac myocytes that are enriched in caveolin-3 (72, 73). Additionally, components of the β-adrenergic receptor signaling cascade are also present in caveolae-enriched membranes (72, 73).

*SCN4B* encodes for NaVβ4, which is an auxiliary β-subunit of the cardiac sodium channel. So far only one mutation (L179F) has been reported in this gene in a Mexican family with multiple affected family members (74). Mutation in this gene was found to result in gain of function of the Nav1.5 current (74).

### LQTS11

In the heart, sympathetic regulation of cardiac action potential duration (APD) is mediated by β-adrenergic receptor (β-AR) activation, which requires assembly of AKAP9 (Yotiao) with the α-subunit (KvLQT1) of the IKs channel. Mutation in AKAP9 causes LQTS11 (75). To date only one mutation, S1570L, in AKAP9 has been reported (75).

### LQTS12

Mutation in the α-1-Syntrophin (SNTN1) gene are causal to LQTS12. Mutation in this gene leads to gain of function of the cardiac sodium channel (Nav1.5), which is the pathological basis of LQTS12 (76).

### LQTS13

G protein-coupled, inwardly rectifying potassium channel subunit (Kir3.4) is encoded by the *KCNJ5* gene. A loss-of-function mutation in this gene could cause LQTS13 (77). So far, only one mutation, G387R, has been described in a Chinese family with nine patients having this mutation. Reduced plasma membrane expression of Kir3.4 was suggested as the pathology of LQTS in the patients.

## Acquired LQTS

In addition to the congenital LQTS, another variant of LQTS known as aLQTS also exist, which is caused by factors and substances that decrease potassium flux and impair the ability of the myocardium to repolarize. Well-recognized conditions are female gender, hypokalemia, and drugs that inhibit cardiac potassium channels (78–80). A number of commonly prescribed drugs could also preferentially bind and block the HERG channel (Kv11.1, a protein encoded by *KCNH2* gene) and predisposes to aLQTS (79, 80). Recently, it was shown that blockade of IKs could also contribute to drug-induced aLQTS, specially when repolarization reserve is compromised (81). Fluoxetine and norfluoxetine were found to suppress the IKs properties, both *in vivo* and *in vitro* and led to marked LQTS (81). Polymorphisms, D85N in minK (gene *KCNE*1), T8A, Q9E in MiRP1 (gene *KCNE2*), which are putative β-sub-units of the IKs and IKr channels, were reported to cause aLQTS (82, 83). Autoimmune LQTS has also been reported in a patient with IgG containing anti-HERG antibodies (84).

## Electrocardiographic Features in the Three Common Forms of Long QT Syndrome

Typical ST-T-wave patterns are present in the majority of genotyped LQTS patients and can be used to identify LQTS1, LQTS2, and possibly LQTS3 genotypes (85, 86).

**Figure 2:**
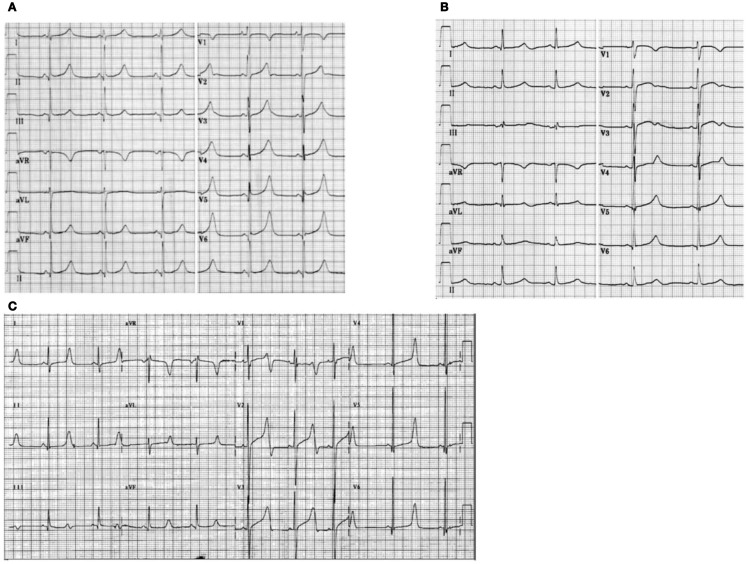
**Electrocardiogram recordings from patients with LQTS1, LQTS2, and LQTS3**. **(A)** 12-lead ECG of an 18-year-old male with a KCNQ1 mutation. The QT interval is prolonged (QTc = ±500 ms). The ST segment has a broad base and relatively large amplitude. Conduction interval is normal (standard calibration). **(B)** 12-lead ECG of a 14-year-old girl with a KCNH2 mutation. The QT interval is prolonged (QTc ± 520 ms). The ST segment is notched in lead V3 and has relatively low amplitude in the extremity leads. Conduction interval is normal (standard calibration). **(C)** 12-lead ECG of a 12-year-old boy with a SCN5A mutation. The QT interval is prolonged (QTc ± 600 ms). The ST segment has a long (almost) iso-electric segment with a large, sharp, and narrow *T* wave. Conduction interval is normal (standard calibration).

The LQTS1 form of the LQTS is associated with a broad *T*-wave without shortening of the QT interval at exercise (Figure 2A). LQTS2 is associated with low-amplitude, often bifid, *T* waves (Figure 2B). LQTS3 is associated with a long iso-electric segment and a narrow-based, tall *T* wave (Figure 2C). Pause dependence of TdP onset in congenital LQTS is genotype-specific, being predominant in LQTS2 but almost absent in LQTS1 (87).

Although patterns may suggest a specific genotype of the LQTS, exceptions have been frequently found.

## Genotype-Phenotype

Long QT syndrome is an autosomal dominant disease. Genotype and phenotype analysis among the heterozygous mutation carriers have been conducted quite extensively in the LQTS1, LQTS2, and LQTS3 patients. During childhood, the risk of cardiac events is significantly higher in LQTS1 males than in LQTS1 females, whereas no significant gender-related differences in risk of cardiac events were observed amongst LQTS2 and LQTS3 patients (88–91). During adulthood (also after age 40), LQTS1 and LQTS2 females could have significantly higher risk of cardiac events than respective males (88–91). In general, lethality of cardiac events seems to be predominant in LQTS3 patients than in LQTS1 and LQTS2 patients (91). Women with LQTS have a reduced risk for cardiac events during pregnancy, but the risk quite increases during the 9-month postpartum period, specially in the women with mutation in the *KCNH2* gene (92).

Sudden cardiac death in children could also be caused by mutations in the cardiac ion channel genes (93–96). About 28% of the children with an unexplained SCD (between 1 and 18 years, mean age: 12.3 ± 3.8 years) were carriers of mutations in LQTS causal genes (97). In SIDS, mutations in SCN5A seemed predominant (98, 99), but, mutations in *KCNQ1, KCNH2, KCNE2*, and *CAV3, SCN4B*, and *SCN3B* were also found (100, 101). Intrauterine fetal deaths were also reported due to defects in cardiac ion channel genes (12, 102).

## Clinical Management of LQTS

Cessation of all drugs that are known to prolong the QT interval and also the correction of electrolyte imbalances and/or precipitating metabolic conditions should be the primary focus while treating (acquired-) LQTS patients. Symptoms in LQTS are often adrenergically mediated, therefore restriction of patients’ participation in athletic activities are generally recommended (24, 25). The mainstay of clinical therapy for the LQTS is β-blockade. Long-acting preparations of propranolol, nadolol, and metoprolol are usually used, and their efficacy in β-blockade is assessed by blunting of the exercise heart rate (e.g., by >20%) (24, 25). Amongst all the β-blockers, propranolol and nadolol are considered superior than metoprolol in symptomatic patients (103). Furthermore, β-blockade can also be used as a prophylactic treatment in silent mutation carriers to reduce SCD (24). In a study by Barsheshet et al. (30), patients with c-loop missense mutations in the KCNQ1 gene exhibited high risk of life-threatening cardiac events and they had significant benefits from the treatment with β-blockers. As women with LQTS2 have an increased risk during the 9-month postpartum period, β-blockers should be prescribed to reduce any cardiac events during this high-risk period (92). An implantable cardioverter–defibrillators (ICDs) can be considered for patients with recurrent syncope despite β-blocker therapy or in patients with high risk for cardiac arrest (e.g., symptomatic LQTS2 and LQTS3 with a documented QTc prolongation). Left cardiac sympathetic denervation (LCSD) is recommended for high risk LQTS patients in whom an ICD is contraindicated or refused and β-blockers are either not effective, not tolerated, not accepted or contraindicated (18, 104). JLNS patients usually have a QTc >500 ms and are also at high risk, β-blockers have insufficient efficacy in such patients in whom an early therapy with ICD was recommended (18, 31).

## LQTS: Saudi Perspective

First report on LQTS from Saudi Arabia was published in 1993 from the Riyadh Armed Forces Hospital (105). Four infants and young children between 6 and 48 months with history of recurrent seizures, from a single family, were diagnosed as LQTS (105). Family history revealed that two-other extended family members had similar episodes of sudden loss of consciousness and three-family members died suddenly (105). In all cases, initial diagnosis was epilepsy (105). Several years later, two sporadic case reports with comparatively severer variant of neonatal LQTS combined with 2:1 AV block have been reported (106, 107). All these published clinical reports were without any genetic findings that could explain the pathophysiology of LQTS in them (105–107).

**Figure 3:**
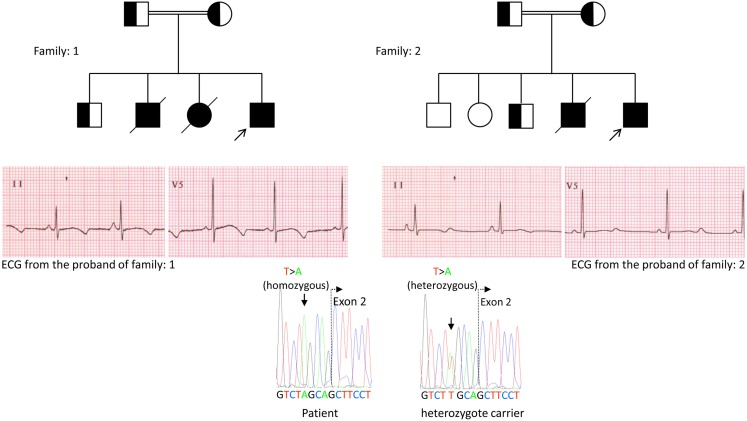
**Pedigree drawing of the family-1 and 2 with autosomal recessive LQTS1, probands are shown with an arrow**. ECGs from the probands in two families are shown in the middle, with a QTc of 557 and 529 ms, respectively. Intronic mutation, c.387 −5 T > A in the *KCNQ1* gene was found in the patients (shown in the bottom). Mutation was shown with an arrow. Non-filled circles and squares are non-carriers for the mutation. Affected individuals are shown as filled circles (female) and squares (male). Half filled squares and circles are individuals with heterozygote mutation. Deceased individuals are indicated by slashes, probands are indicated by an arrow and consanguineous marriage is indicated by = Exon − intron boundary is shown by a dotted line with arrow pointing toward exon.

**Figure 4:**
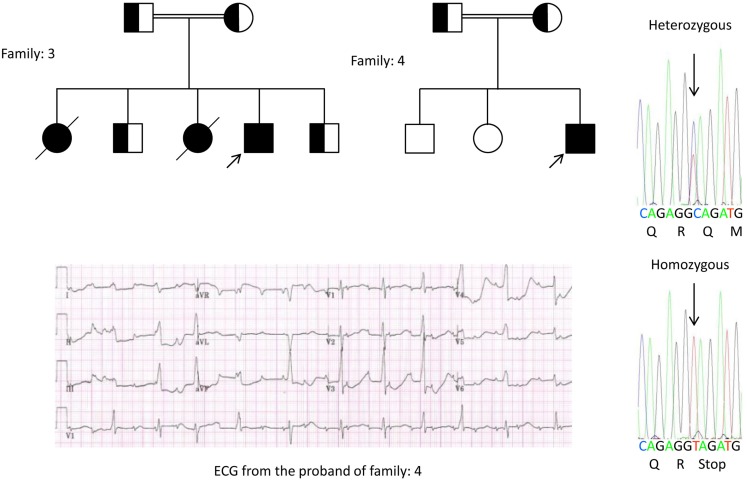
**Pedigree drawing of the family-3 and 4 with autosomal recessive LQTS2**. ECG of the proband from family-4 is shown below the pedigree drawing, which shows sinus tachycardia, almost complete AV block, wide complex escape rhythm with very long QTc intervals (QT >600 ms). Mutation, c.3208 C > T (p.Q1070X) in the *KCNH2* gene was found in the patients (shown on the right), marked with an arrow.

**Figure 5:**
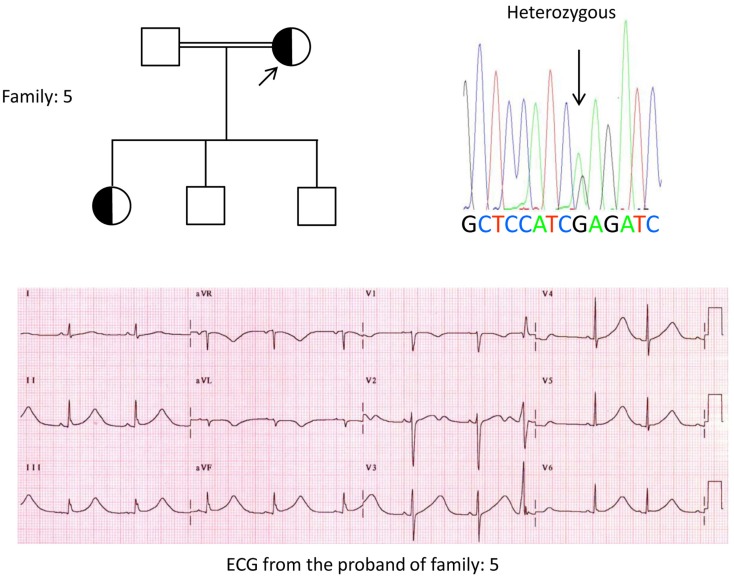
**Top left: pedigree of the family-5**. Affected individual is shown by filled circle (female) Proband is indicated by an arrow. 12-lead ECG of the proband (I:2). ECG shows a high peaked, broad based, large amplitude *T* wave, QTc interval is 580 ms. Screening of the KCNH2 gene shows substitution of nucleotide “G” for an “A” (c.2362G > A, arrow marked), which leads to amino acid substitution, p.E788K.

We, for the first time, have reported genetic defects as a pathological basis of LQTS in similar patients from Saudi Arabia. We have investigated six-Saudi families with history of syncope and sudden unexplained deaths of fetus, neonates and children (12–14). Autosomal recessive LQTS1 was diagnosed in children from two families (Figure 3). Autosomal recessive LQTS2 was diagnosed in two families (Figure 4). In one family, a female patient was diagnosed with autosomal dominant LQTS2 (Figure 5), the patient had syncopal attacks during postpartum recovery period at the hospital, which is very common in *KCNH2* mutation carrier women. In all our patients, identification of pathogenic mutations in the LQTS causal cardiac ion channel genes led to a confirmed clinical diagnosis, which were misdiagnosed as epileptic convulsions before being referred to us (13, 14) Recently, Shinwari et al. (15) from King Faisal Specialist Hospital and Research Centre reported a LQTS1 causal KCNQ1 mutation, H258P, in a large family with 12 affected individuals. Only two carriers were symptomatic, their QTc were >500 ms, and β-blockers suppressed the clinical symptoms in one patient and the second symptomatic patient required an ICD.

**Figure 6:**
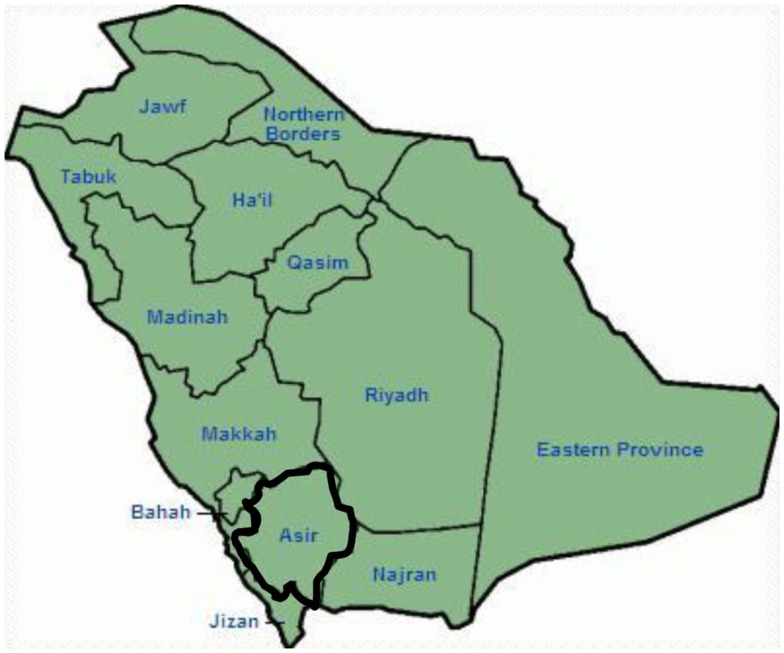
**Map of Saudi Arabia (courtesy: Wikipedia)**. Assir region is marked with thick line, where we have found the founder mutations in *KCNQ1* and *KCNH2* genes, described in family 1–4.

Genetic and clinical findings in our study (12–14) from Saudi Arabia are quite intriguing for several reasons: (1) In total, we have investigated six-families, among them four were homozygous/compound heterozygous for the mutations and the mutations originated from an ancestral source; (2) All the mutations in LQTS were novel, reported only in these Arab families; (3) Due to the homozygosity or compound heterozygosity for the mutations, clinical phenotypes were also severe in our studied families (12–14). We suggested that the genetic and phenotypic observations stemmed from the extreme high rate of consanguineous marriages in Saudi Arabia (108, 109). Our study provided the first scientific evidence about the role of consanguinity in exerting a pivotal role in unexplained cardiac arrhythmias and SCDs in children and adolescents in Saudi Arabia (12–14). We have also shown that the *KCNQ1* mutation c.387 −5 T > A (NM_000218) (Figure 3) had spread in the Assir province of Saudi Arabia from a common ancestor during several generations due to high incidence of consanguineous marriages [see the map, Figure 6; (12–14)]. So far, this is the most common LQTS1 causal mutation in Saudi Arabian population, which was also observed at King Faisal Specialist Hospital and at Khamis Mashayt Military Hospital (unpublished). A similar result was also obtained for LQTS2 causal mutation, c.3208 C > T (p.Q1070X) (Figure 4) in the *KCNH2* gene (12, 14), which is also a founder mutation in Saudi Arabia and segregated during many generations in the Assir region (see the map, Figure 6). We would predict that there exist a considerable number of individuals with the mentioned ancestral/founder mutations (both in KCNQ1 and KCNH2 and other genes) in this region and also in the big cities like Riyadh, Jeddah and Dammam due to urban migration. More founder or ancestral mutations pathogenic to LQTS are very much envisaged in other provinces of Saudi Arabia due to high rate of consanguineous marriages.

As LQTS is an autosomal dominant disease, we expected to observe predominantly heterozygous mutation carrier patients. But in our study, we mainly identified patients with recessive mutations (12–14). Clearly, these patients become first symptomatic and, were predominantly referred to Prince Sultan Cardiac Centre, bringing them under our attention. Nevertheless, findings from our investigations implies to the fact that recessive LQTS could have fatal clinical phenotypes in children, and they are not uncommon in Saudi Arabia (12–14). As the heterozygous mutation carriers are also susceptible to develop arrhythmia and its complications, concerted initiative should be taken to bring the local general physicians, cardiologists, and clinical geneticists in a common platform to identify the individuals at risk. Further, at the moment, we have also no genetic information for other familial arrhythmia disorders, e.g., CPVT, SQTS, BrS, AF, etc. Specialized cardiogenetic centers should take the initiative to search for the genetic defects, mutations, and perform genotype-phenotype studies in all forms of hereditary arrhythmias. It also should be kept in consideration that not all genetic arrhythmias would have a family history, as in many cases arrhythmia causal mutation are *de novo* in origin, i.e., the proband is the first patient in that family with the mutation and he/she is the source to transmit the mutation in downstream generations (110, 111). Due to the very high rate of consanguineous marriages in Saudi Arabia, we expect a lot more founder mutations exert a crucial role in congenital arrhythmias in this country. Identifying these founder mutations should be our first and foremost task, which would facilitate us in developing an effective premarital and also pre-symptomatic genetic counseling in this country. Mutations in LQTS causal genes could confer variability in clinical penetrance, in one extreme, some carriers could be presumably completely healthy, but some carriers could have their first manifestation of the disease as syncope or sudden death. Sudden death of a young child or adult poses a great deal of psychological and emotional burden on the family, screening of the carriers are essential as there exist simple medication, e.g., β-blockers (also behavior modification), which could very effectively prevent the carriers from the fatal consequences of arrhythmia and SCDs. Individuals with homozygous mutations in the LQTS causal genes could have severe morbidity and extreme high rate of mortality including fetal brady-tachyarrhythmias, and in many cases miscarriages, spontaneous abortions in the pregnant mothers (12–14).

Not much research has also been conducted in Saudi Arabia about the prevalence of various SNPs in the arrhythmia linked genes and also in the genes that regulate them. Splawski et al. (112) described a common S1103Y variant in the SCN5A gene associated with arrhythmia in African-Americans. The variant allele termed Y1103 is responsible for accelerating channel activation, thereby enhancing the probability of cardiac arrhythmias in persons of African descent (52, 112). K393N is a variant in KCNQ1 gene reported in LQTS1 patients in USA, but, in the Arab population we have detected this variant in 2% of individuals (unpublished data). Whether, K393N variant in *KCNQ1* gene in Arabs is comparable to the S1103Y (*SCN5A*) variant in African-Americans or the D85N (*KCNE1*) variant in the Japanese population could also merit investigation (59).

## Conflict of Interest Statement

The authors declare that the research was conducted in the absence of any commercial or financial relationships that could be construed as a potential conflict of interest.

## References

[1] Bhuiyan ZA Clinical and Genetic Spectrum of Hereditary Cardiac Arrhythmia Syndromes. Amsterdam: Amsterdam University (2009).

[2] Priori SG Aliot E Blømstrom-Lundqvist C Bossaert L Breithardt G Brugada P Task force on sudden cardiac death, European society of cardiology. Europace (2002) 4:3–18 10.1053/eupc.2001.0214 11858152

[3] Wang Q Shen J Splawski I Atkinson D Li Z Robinson JL SCN5A mutations associated with an inherited cardiac arrhythmia, long QT syndrome. Cell (1995) 80:805–11 10.1016/0092-8674(95)90359-3 7889574

[4] Curran ME Splawski I Timothy KW Vincent GM Green ED Keating MT A molecular basis for cardiac arrhythmia: HERG mutations cause long QT syndrome. Cell (1995) 80:795–803 10.1016/0092-8674(95)90358-5 7889573

[5] Wang Q Curran ME Splawski I Burn TC Millholland JM VanRaay TJ Positional cloning of a novel potassium channel gene: KVLQT1 mutations cause cardiac arrhythmias. Nat Genet (1996) 12:17–23 10.1038/ng0196-17 8528244

[6] Splawski I Tristani-Firouzi M Lehmann MH Sanguinetti MC Keating MT Mutations in the hminK gene cause long QT syndrome and suppress IKs function. Nat Genet (1997) 17:338–40 10.1038/ng1197-338 9354802

[7] Sanguinetti MC Jiang C Curran ME Keating MT A mechanistic link between an inherited and an acquired cardiac arrhythmia: HERG encodes the IKr potassium channel. Cell (1995) 81:299–307 10.1016/0092-8674(95)90340-2 7736582

[8] Neyroud N Tesson F Denjoy I Leibovici M Donger C Barhanin J A novel mutation in the potassium channel gene KVLQT1 causes the Jervell and Lange-Nielsen cardioauditory syndrome. Nat Genet (1997) 15:186–9 10.1038/ng0297-186 9020846

[9] Kucera JP Rohr S Rudy Y Localization of sodium channels in intercalated disks modulates cardiac conduction. Circ Res (2002) 91:1176–82 10.1161/01.RES.0000046237.54156.0A 12480819 PMC1888562

[10] Oxford EM Musa H Maass K Coombs W Taffet SM Delmar M Connexin43 remodeling caused by inhibition of plakophilin-2 expression in cardiac cells. Circ Res (2007) 101:703–11 10.1161/CIRCRESAHA.107.154252 17673670

[11] Rohr S Molecular crosstalk between mechanical and electrical junctions at the intercalated disc. Circ Res (2007) 101:637–9 10.1161/CIRCRESAHA.107.161901 17901364

[12] Bhuiyan ZA Momenah TS Gong Q Amin AS Ghamdi SA Carvalho JS Recurrent intrauterine fetal loss due to near absence of HERG: clinical and functional characterization of a homozygous nonsense HERG Q1070X mutation. Heart Rhythm (2008) 5:553–61 10.1016/j.hrthm.2008.01.020 18362022 PMC2682734

[13] Bhuiyan ZA Momenah TS Amin TS Al-Khadra AS Alders M Wilde AAM An Intronic mutation leading to incomplete skipping of exon-2 in KCNQ1 rescues hearing in Jervell and Lange-Nielsen syndrome. Prog Biophys Mol Biol (2008) 98:319–27 10.1016/j.pbiomolbio.2008.10.004 19027783

[14] Bhuiyan ZA Al-Shahrani S Al-Khadra AS Al-Ghamdi S Al-Kalaf K Mannens MMAM Clinical and genetic analysis of long QT syndrome in children from six families in Saudi Arabia: are they different? Pediatr Cardiol (2009) 30:490–501 10.1007/s00246-008-9377-y 19184172

[15] Shinwari ZM Al-Hazzani A Dzimiri N Tulbah S Mallawi Y Al-Fayyadh M Identification of a novel KCNQ1 mutation in a large Saudi family with long QT syndrome: clinical consequences and preventive implications. Clin Genet (2013) 83:370–4 10.1111/j.1399-0004.2012.01914.x 22708720

[16] Schwartz PJ Stramba-Badiale M Crotti L Pedrazzini M Besana A Bosi G Prevalence of the congenital long-QT syndrome. Circulation (2009) 120:1761–7 10.1161/CIRCULATIONAHA.109.863209 19841298 PMC2784143

[17] Schwartz PJ Moss AJ Vincent GM Crampton RS Diagnostic criteria for the long QT syndrome. An update. Circulation (1993) 88:782–4 10.1161/01.CIR.88.2.782 8339437

[18] Priori SG Wilde AA Horie M Cho Y Behr ER Berul C Executive summary: HRS/EHRA/APHRS consensus statement on the diagnosis and management of patients with inherited primary arrhythmia syndromes. Europace (2013) 15:1389–406 10.1093/europace/eut272 23994779

[19] Liu JF Jons C Moss AJ McNitt S Peterson DR Qi M Syndrome Registry. Risk factors for recurrent syncope and subsequent fatal or near-fatal events in children and adolescents with long QT syndrome. J Am Coll Cardiol (2011) 57:941–50 10.1016/j.jacc.2010.10.025 21329841 PMC3052409

[20] Splawski I Shen J Timothy KW Lehmann MH Priori S Robinson JL Spectrum of mutations in long-QT syndrome genes. KVLQT1, HERG, SCN5A, KCNE1, and KCNE2. Circulation (2000) 102:1178–85 10.1161/01.CIR.102.10.1178 10973849

[21] Westenskow P Splawski I Timothy KW Keating MT Sanguinetti MC Compound mutations: a common cause of severe long-QT syndrome. Circulation (2004) 109:1834–41 10.1161/01.CIR.0000125524.34234.13 15051636

[22] Mullally J Goldenberg I Moss AJ Lopes CM Ackerman MJ Zareba W Risk of life-threatening cardiac events among patients with long QT syndrome and multiple mutations. Heart Rhythm (2013) 10:378–82 10.1016/j.hrthm.2012.11.006 23174487 PMC3690288

[23] Napolitano C Priori SG Schwartz PJ Bloise R Ronchetti E Nastoli J Genetic testing in the long QT syndrome: development and validation of an efficient approach to genotyping in clinical practice. JAMA (2005) 294:2975–80 10.1001/jama.294.23.2975 16414944

[24] Roden DM Clinical practice. Long-QT syndrome. N Engl J Med (2008) 358:169–76 10.1056/NEJMcp0706513 18184962

[25] Schwartz PJ Priori SG Spazzolini C Moss AJ Vincent GM Napolitano C Genotype-phenotype correlation in the long-QT syndrome: gene-specific triggers for life-threatening arrhythmias. Circulation (2001) 103:89–95 10.1161/01.CIR.103.1.89 11136691

[26] Ackerman MJ Tester DJ Porter CJ Swimming, a gene-specific arrhythmogenic trigger for inherited long QT syndrome. Mayo Clin Proc (1999) 74:1088–94 10.4065/74.11.1088 10560595

[27] Kapa S Tester DJ Salisbury BA Harris-Kerr C Pungliya MS Alders M Genetic testing for long-QT syndrome: distinguishing pathogenic mutations from benign variants. Circulation (2009) 120:1752–60 10.1161/CIRCULATIONAHA.109.863076 19841300 PMC3025752

[28] Moss AJ Shimizu W Wilde AA Towbin JA Zareba W Robinson JL Clinical aspects of type-1 long-QT syndrome by location, coding type, and biophysical function of mutations involving the KCNQ1 gene. Circulation (2007) 115:2481–9 10.1161/CIRCULATIONAHA.106.665406 17470695 PMC3332528

[29] Amin AS Giudicessi JR Tijsen AJ Spanjaart AM Reckman YJ Klemens CA Variants in the 3′ untranslated region of the KCNQ1-encoded Kv7.1 potassium channel modify disease severity in patients with type 1 long QT syndrome in an allele-specific manner. Eur Heart J (2012) 33:714–23 10.1093/eurheartj/ehr473 22199116 PMC3303714

[30] Barsheshet A Goldenberg I O-Uchi J Moss AJ Jons C Shimizu W Mutations in cytoplasmic loops of the KCNQ1 channel and the risk of life threatening events: implications for mutation-specific response to β-blocker therapy in type 1 long-QT syndrome. Circulation (2012) 125:1988–96 10.1161/CIRCULATIONAHA.111.048041 22456477 PMC3690492

[31] Schwartz PJ Spazzolini C Crotti L Bathen J Amlie JP Timothy K The Jervell and Lange-Nielsen syndrome: natural history, molecular basis, and clinical outcome. Circulation (2006) 113:783–90 10.1161/CIRCULATIONAHA.105.592899 16461811

[32] Bhuiyan ZA Wilde AA IKs in heart and hearing, the ear can do with less than the heart. Circ Cardiovasc Genet (2013) 6:141–3 10.1161/CIRCGENETICS.113.000143 23591039

[33] Wollnik B Schroeder BC Kubisch C Esperer HD Wieacker P Jentsch TJ Pathophysiological mechanisms of dominant and recessive KVLQT1 K+ channel mutations found in inherited cardiac arrhythmias. Hum Mol Genet (1997) 6:1943–9 10.1093/hmg/6.11.1943 9302275

[34] Wilde AA Jongbloed RJ Doevendans PA Düren DR Hauer RN van Langen IM Auditory stimuli as a trigger for arrhythmic events differentiate HERG-related (LQTS2) patients from KVLQT1-related patients (LQTS1). J Am Coll Cardiol (1999) 33:327–32 10.1016/S0735-1097(98)00578-6 9973011

[35] Moss AJ Zareba W Kaufman ES Gartman E Peterson DR Benhorin J Increased risk of arrhythmic events in long-QT syndrome with mutations in the pore region of the human ether-a-go-go-related gene potassium channel. Circulation (2002) 105:794–9 10.1161/hc0702.105124 11854117

[36] Lupoglazoff JM Denjoy I Villain E Fressart V Simon F Bozio A Long QT syndrome in neonates: conduction disorders associated with HERG mutations and sinus bradycardia with KCNQ1 mutations. J Am Coll Cardiol (2004) 43:826–30 10.1016/j.jacc.2003.09.049 14998624

[37] Chevalier P Bellocq C Millat G Piqueras E Potet F Schott JJ Torsades de pointes complicating atrioventricular block: evidence for a genetic predisposition. Heart Rhythm (2007) 4:170–4 10.1016/j.hrthm.2006.10.004 17275752

[38] Hoorntje T Alders M van Tintelen P van der Lip K Sreeram N van der Wal A Homozygous premature truncation of the HERG protein: the human HERG knockout. Circulation (1999) 100:1264–7 10.1161/01.CIR.100.12.1264 10491368

[39] Piippo K Laitinen P Swan H Toivonen L Viitasalo M Pasternack M Homozygosity for a HERG potassium channel mutation causes a severe form of long QT syndrome: identification of an apparent founder mutation in the Finns. J Am Coll Cardiol (2000) 35:1919–25 10.1016/S0735-1097(00)00636-7 10841244

[40] Johnson WH Jr Yang P Yang T Lau YR Mostella BA Wolff DJ Clinical, genetic, and biophysical characterization of a homozygous HERG mutation causing severe neonatal long QT syndrome. Pediatr Res (2003) 53:744–8 10.1203/01.PDR.0000059750.17002.B6 12621127

[41] Ackerman MJ Tester DJ Jones GS Will ML Burrow CR Curran ME Ethnic differences in cardiac potassium channel variants: implications for genetic susceptibility to sudden cardiac death and genetic testing for congenital long QT syndrome. Mayo Clin Proc (2003) 78:1479–87 10.4065/78.12.1479 14661677

[42] Crotti L Lundquist AL Insolia R Pedrazzini M Ferrandi C De Ferrari GM KCNH2-K897T is a genetic modifier of latent congenital long-QT syndrome. Circulation (2005) 112:1251–8 10.1161/CIRCULATIONAHA.105.549071 16116052

[43] Nof E Cordeiro JM Pérez GJ Scornik FS Calloe K Love B A common single nucleotide polymorphism can exacerbate long-QT type 2 syndrome leading to sudden infant death. Circ Cardiovasc Genet (2010) 3:199–206 10.1161/CIRCGENETICS.109.898569 20181576 PMC2858238

[44] Bezzina C Veldkamp MW van Den Berg MP Postma AV Rook MB Viersma JW A single Na(+) channel mutation causing both long-QT and Brugada syndromes. Circ Res (1999) 85:1206–13 10.1161/01.RES.85.12.1206 10590249

[45] Kyndt F Probst V Potet F Demolombe S Chevallier JC Baro I Novel SCN5A mutation leading either to isolated cardiac conduction defect or Brugada syndrome in a large French family. Circulation (2001) 104:3081–6 10.1161/hc5001.100834 11748104

[46] Makita N Behr E Shimizu W Horie M Sunami A Crotti L The E1784K mutation in SCN5A is associated with mixed clinical phenotype of type 3 long QT syndrome. J Clin Invest (2008) 118:2219–29 10.1172/JCI34057 18451998 PMC2350431

[47] Keller DI Acharfi S Delacrétaz E Benammar N Rotter M Pfammatter JP A novel mutation in SCN5A, delQKP 1507-1509, causing long QT syndrome: role of Q1507 residue in sodium channel inactivation. J Mol Cell Cardiol (2003) 35:1513–21 10.1016/j.yjmcc.2003.08.007 14654377

[48] Priori SG Schwartz PJ Napolitano C Bloise R Ronchetti E Grillo M Risk stratification in the long-QT syndrome. N Engl J Med (2003) 348:1866–74 10.1056/NEJMoa022147 12736279

[49] Lupoglazoff JM Cheav T Baroudi G Berthet M Denjoy I Cauchemez B Homozygous SCN5A mutation in long-QT syndrome with functional two-to-one atrioventricular block. Circ Res (2001) 89:E16–21 10.1161/hh1401.095087 11463728

[50] Shi R Zhang Y Yang C Huang C Zhou X Qiang H The cardiac sodium channel mutation delQKP 1507-1509 is associated with the expanding phenotypic spectrum of LQT3, conduction disorder, dilated cardiomyopathy, and high incidence of youth sudden death. Europace (2008) 10:1329–35 10.1093/europace/eun202 18697752 PMC2573028

[51] Zumhagen S Veldkamp MW Stallmeyer B Baartscheer A Eckardt L Paul M A heterozygous deletion mutation in the cardiac sodium channel gene SCN5A with loss- and gain-of-function characteristics manifests as isolated conduction disease, without signs of Brugada or long QT syndrome. PLoS One (2013) 8:e67963 10.1371/journal.pone.0067963 23840796 PMC3695936

[52] Plant LD Bowers PN Liu Q Morgan T Zhang T State MW A common cardiac sodium channel variant associated with sudden infant death in African Americans, SCN5A S1103Y. J Clin Invest (2006) 116:430–5 10.1172/JCI25618 16453024 PMC1359045

[53] Mohler PJ Schott JJ Gramolini AO Dilly KW Guatimosim S duBell WH Ankyrin-B mutation causes type 4 long-QT cardiac arrhythmia and sudden cardiac death. Nature (2003) 421:634–9 10.1038/nature01335 12571597

[54] Schott JJ Charpentier F Peltier S Foley P Drouin E Bouhour JB Mapping of a gene for long QT syndrome to chromosome 4q25-27. Am J Hum Genet (1995) 57:1114–22 7485162 PMC1801360

[55] Barhanin J Lesage F Guillemare E Fink M Lazdunski M Romey G K(V)LQT1 and lsK (minK) proteins associate to form the I(Ks) cardiac potassium current. Nature (1996) 384:78–80 10.1038/384078a0 8900282

[56] Sanguinetti MC Curran ME Zou A Shen J Spector PS Atkinson DL Coassembly of K(V)LQT1 and minK (IsK) proteins to form cardiac I(Ks) potassium channel. Nature (1996) 384:80–3 10.1038/384080a0 8900283

[57] Schulze-Bahr E Wang Q Wedekind H Haverkamp W Chen Q Sun Y KCNE1 mutations cause Jervell and Lange-Nielsen syndrome. Nat Genet (1997) 17:267–8 10.1038/ng1197-267 9354783

[58] Duggal P Vesely MR Wattanasirichaigoon D Villafane J Kaushik V Beggs AH Mutation of the gene for IKs associated with both Jervell and Lange-Nielsen and Romano-Ward forms of long-QT syndrome. Circulation (1998) 97:142–6 10.1186/1471-2350-9-24 9445165

[59] Nishio Y Makiyama T Itoh H Sakaguchi T Ohno S Gong YZ D85N, a KCNE1 polymorphism, is a disease-causing gene variant in long QT syndrome. J Am Coll Cardiol (2009) 54:812–9 10.1016/j.jacc.2009.06.005 19695459

[60] Paulussen AD Gilissen RA Armstrong M Doevendans PA Verhasselt P Smeets HJ Genetic variations of KCNQ1, KCNH2, SCN5A, KCNE1, and KCNE2 in drug-induced long QT syndrome patients. Mol Med (2004) 82:182–8 10.1007/s00109-003-0522-z 14760488

[61] Abbott GW Sesti F Splawski I Buck ME Lehmann MH Timothy KW MiRP1 forms IKr potassium channels with HERG and is associated with cardiac arrhythmia. Cell (1999) 97:175–87 10.1016/S0092-8674(00)80728-X 10219239

[62] Gordon E Panaghie G Deng L Bee KJ Roepke TK Krogh-Madsen T A KCNE2 mutation in a patient with cardiac arrhythmia induced by auditory stimuli and serum electrolyte imbalance. Cardiovasc Res (2008) 77:98–106 10.1093/cvr/cvm030 18006462

[63] Plaster NM Tawil R Tristani-Firouzi M Canún S Bendahhou S Tsunoda A Mutations in Kir2.1 cause the developmental and episodic electrical phenotypes of Andersen’s syndrome. Cell (2001) 105:511–9 10.1016/S0092-8674(01)00342-7 11371347

[64] Kubo Y Baldwin TJ Jan YN Jan LY Primary structure and functional expression of a mouse inward rectifier potassium channel. Nature (1993) 362:127–33 10.1038/362127a0 7680768

[65] Raab-Graham KF Radeke CM Vandenberg CA Molecular cloning and expression of a human heart inward rectifier potassium channel. Neuroreport (1994) 5:2501–5 10.1097/00001756-199412000-00024 7696590

[66] Splawski I Timothy KW Sharpe LM Decher N Kumar P Bloise R Ca(V)1.2 calcium channel dysfunction causes a multisystem disorder including arrhythmia and autism. Cell (2004) 119:19–31 10.1016/j.cell.2004.09.011 15454078

[67] Splawski I Timothy KW Decher N Kumar P Sachse FB Beggs AH Severe arrhythmia disorder caused by cardiac L-type calcium channel mutations. Proc Natl Acad Sci U S A (2005) 102:8089–96 10.1073/pnas.0502506102 15863612 PMC1149428

[68] Gillis J Burashnikov E Antzelevitch C Blaser S Gross G Turner L Long QT, syndactyly, joint contractures, stroke and novel CACNA1C mutation: expanding the spectrum of Timothy syndrome. Am J Med Genet A (2012) 158A:182–7 10.1002/ajmg.a.34355 22106044 PMC3319791

[69] Dufendach KA Giudicessi JR Boczek NJ Ackerman MJ Maternal mosaicism confounds the neonatal diagnosis of type 1 Timothy syndrome. Pediatrics (2013) 131:e1991–5 10.1542/peds.2012-2941 23690510 PMC3666110

[70] Vatta M Ackerman MJ Ye B Makielski JC Ughanze EE Taylor EW Mutant caveolin-3 induces persistent late sodium current and is associated with long-QT syndrome. Circulation (2006) 114:2104–12 10.1161/CIRCULATIONAHA.106.635268 17060380

[71] Cronk LB Ye B Kaku T Tester DJ Vatta M Makielski JC Novel mechanism for sudden infant death syndrome: persistent late sodium current secondary to mutations in caveolin-3. Heart Rhythm (2007) 4:161–6 10.1016/j.hrthm.2006.11.030 17275750 PMC2836535

[72] Engelman JA Zhang X Galbiati F Volonte D Sotgia F Pestell RG Molecular genetics of the caveolin gene family: implications for human cancers, diabetes, Alzheimer disease, and muscular dystrophy. Am J Hum Genet (1998) 63:1578–87 10.1086/302172 9837809 PMC1377628

[73] Balijepalli RC Foell JD Hall DD Hell JW Kamp TJ Localization of cardiac L-type Ca(2+) channels to a caveolar macromolecular signaling complex is required for beta(2)-adrenergic regulation. Proc Natl Acad Sci U S A (2006) 103:7500–5 10.1073/pnas.0503465103 16648270 PMC1564282

[74] Medeiros-Domingo A Kaku T Tester DJ Iturralde-Torres P Itty A Ye B SCN4B-encoded sodium channel beta4 subunit in congenital long-QT syndrome. Circulation (2007) 116:134–42 10.1161/CIRCULATIONAHA.106.659086 17592081 PMC3332546

[75] Chen L Marquardt ML Tester DJ Sampson KJ Ackerman MJ Kass RS Mutation of an A-kinase-anchoring protein causes long-QT syndrome. Proc Natl Acad Sci U S A (2007) 104:20990–5 10.1073/pnas.0710527105 18093912 PMC2409254

[76] Wu G Ai T Kim JJ Mohapatra B Xi Y Li Z Alpha-1-syntrophin mutation and the long QT Syndrome: a disease of sodium channel disruption. Circ Arrhythm Electrophysiol (2008) 1:193–201 10.1161/CIRCEP.108.769224 19684871 PMC2726717

[77] Yang Y Yang Y Liang B Liu J Li J Grunnet M Identification of a Kir3.4 mutation in congenital long QT syndrome. Am J Hum Genet (2010) 86:872–80 10.1016/j.ajhg.2010.04.017 20560207 PMC3032079

[78] Roden DM Mechanisms and management of proarrhythmia. Am J Cardiol (1998) 82:49I–57I 10.1016/S0002-9149(98)00472-X 9737654

[79] Mitcheson JS Chen J Lin M Culberson C Sanguinetti MC A structural basis for drug-induced long QT syndrome. Proc Natl Acad Sci U S A (2000) 97:12329–33 10.1073/pnas.210244497 11005845 PMC17341

[80] Kannankeril PJ Roden DM Drug-induced long QT and torsade de pointes: recent advances. Curr Opin Cardiol (2007) 22:39–43 10.1097/HCO.0b013e32801129eb 17143043

[81] Veerman CC Verkerk AO Blom MT Klemens CA Langendijk PN van Ginneken AC Slow delayed rectifier potassium current blockade contributes importantly to drug-induced long QT syndrome. Circ Arrhythm Electrophysiol (2013) 6:1002–9 10.1161/CIRCEP.113.000239 23995305

[82] Sesti F Abbott GW Wei J Murray KT Saksena S Schwartz PJ A common polymorphism associated with antibiotic-induced cardiac arrhythmia. Proc Natl Acad Sci U S A (2000) 97:10613–8 10.1073/pnas.180223197 10984545 PMC27073

[83] Kääb S Crawford DC Sinner MF Behr ER Kannankeril PJ Wilde AA A large candidate gene survey identifies the KCNE1 D85N polymorphism as a possible modulator of drug-induced torsades de pointes. Circ Cardiovasc Genet (2012) 5:91–9 10.1161/CIRCGENETICS.111.960930 22100668 PMC3288202

[84] Nakamura K Katayama Y Kusano KF Haraoka K Tani Y Nagase S Anti-KCNH2 antibody-induced long QT syndrome: novel acquired form of long QT syndrome. J Am Coll Cardiol (2007) 50:1808–9 10.1016/j.jacc.2007.07.037 17964047

[85] Moss AJ Zareba W Benhorin J Locati EH Hall WJ Robinson JL ECG T-wave patterns in genetically distinct forms of the hereditary long QT syndrome. Circulation (1995) 92:2929–34 10.1161/01.CIR.92.10.2929 7586261

[86] Zhang L Timothy KW Vincent GM Lehmann MH Fox J Giuli LC Spectrum of ST-T-wave patterns and repolarization parameters in congenital long-QT syndrome: ECG findings identify genotypes. Circulation (2000) 102:2849–55 10.1161/01.CIR.102.23.2849 11104743

[87] Tan HL Bardai A Shimizu W Moss AJ Schulze-Bahr E Noda T Genotype-specific onset of arrhythmias in congenital long-QT syndrome: possible therapy implications. Circulation (2006) 114:2096–103 10.1161/CIRCULATIONAHA.106.642694 17088455

[88] Locati EH Zareba W Moss AJ Schwartz PJ Vincent GM Lehmann MH Age- and sex-related differences in clinical manifestations in patients with congenital long-QT syndrome: findings from the International LQTS Registry. Circulation (1998) 97:2237–44 10.1161/01.CIR.97.22.2237 9631873

[89] Zareba W Moss AJ Schwartz PJ Vincent GM Robinson JL Priori SG Influence of genotype on the clinical course of the long-QT syndrome. International Long-QT Syndrome Registry Research Group. N Engl J Med (1998) 339:960–5 10.1056/NEJM199810013391404 9753711

[90] Goldenberg I Moss AJ Bradley J Polonsky S Peterson DR McNitt S Long-QT syndrome after age 40. Circulation (2008) 117:2192–201 10.1161/CIRCULATIONAHA.107.729368 18427134

[91] Zareba W Moss AJ Locati EH Lehmann MH Peterson DR Hall WJ Syndrome Registry. Modulating effects of age and gender on the clinical course of long QT syndrome by genotype. J Am Coll Cardiol (2003) 42:103–9 10.1016/S0735-1097(03)00554-0 12849668

[92] Seth R Moss AJ McNitt S Zareba W Andrews ML Qi M Long QT syndrome and pregnancy. J Am Coll Cardiol (2007) 49:1092–8 10.1016/j.jacc.2006.09.054 17349890

[93] Schwartz PJ Priori SG Dumaine R Napolitano C Antzelevitch C Stramba-Badiale M A molecular link between the sudden infant death syndrome and the long-QT syndrome. N Engl J Med (2000) 343:262–7 10.1056/NEJM200007273430405 10911008

[94] Schwartz PJ Priori SG Bloise R Napolitano C Ronchetti E Piccinini A Molecular diagnosis in a child with sudden infant death syndrome. Lancet (2001) 358:1342–3 10.1016/S0140-6736(01)06450-9 11684219

[95] Christiansen M Tønder N Larsen LA Andersen PS Simonsen H Oyen N Mutations in the HERG K+-ion channel: a novel link between long QT syndrome and sudden infant death syndrome. Am J Cardiol (2005) 95:433–4 10.1016/j.amjcard.2004.09.054 15670565

[96] Tester DJ Ackerman MJ Postmortem long QT syndrome genetic testing for sudden unexplained death in the young. J Am Coll Cardiol (2007) 49:240–6 10.1016/j.jacc.2006.10.010 17222736

[97] Hofman N Tan HL Clur SA Alders M van Langen IM Wilde AA Contribution of inherited heart disease to sudden cardiac death in childhood. Pediatrics (2007) 120:e967–73 10.1542/peds.2006-3751 17908752

[98] Wedekind H Smits JP Schulze-Bahr E Arnold R Veldkamp MW Bajanowski T De novo mutation in the SCN5A gene associated with early onset of sudden infant death. Circulation (2001) 104:1158–64 10.1161/hc3501.095361 11535573

[99] Wang DW Desai RR Crotti L Arnestad M Insolia R Pedrazzini M Cardiac sodium channel dysfunction in sudden infant death syndrome. Circulation (2007) 115:368–76 10.1161/CIRCULATIONAHA.106.646513 17210841

[100] Van Norstrand DW Valdivia CR Tester DJ Ueda K London B Makielski JC Molecular and functional characterization of novel glycerol-3-phosphate dehydrogenase 1 like gene (GPD1-L) mutations in sudden infant death syndrome. Circulation (2007) 116:2253–9 10.1161/CIRCULATIONAHA.107.704627 17967976 PMC3332545

[101] Tan BH Pundi KN Van Norstrand DW Valdivia CR Tester DJ Medeiros-Domingo A Sudden infant death syndrome-associated mutations in the sodium channel beta subunits. Heart Rhythm (2010) 7:771–8 10.1016/j.hrthm.2010.01.032 20226894 PMC2909680

[102] Miller TE Estrella E Myerburg RJ Garcia de Viera J Moreno N Rusconi P Recurrent third-trimester fetal loss and maternal mosaicism for long-QT syndrome. Circulation (2004) 109:3029–34 10.1161/01.CIR.0000130666.81539.9E 15184283

[103] Chockalingam P Crotti L Girardengo G Johnson JN Harris KM van der Heijden JF Not all beta-blockers are equal in the management of long QT syndrome types 1 and 2: higher recurrences of events under Metoprolol. J Am Coll Cardiol (2012) 60:2092–6 10.1016/j.jacc.2012.07.046 23083782 PMC3515779

[104] Schwartz PJ Priori SG Cerrone M Spazzolini C Odero A Napolitano C Left cardiac sympathetic denervation in the management of high-risk patients affected by the long-QT syndrome. Circulation (2004) 109:1826–33 10.1161/01.CIR.0000125523.14403.1E 15051644

[105] Singh B al Shahwan SA Habbab MA al Deeb SM Biary N Idiopathic long QT syndrome: asking the right question. Lancet (1993) 341:741 10.1016/0140-6736(93)90501-7 8095637

[106] Gorgels AP Al Fadley F Zaman L Kantoch MJ Al Halees Z The long QT syndrome with impaired atrioventricular conduction: a malignant variant in infants. J Cardiovasc Electrophysiol (1998) 9:1225–32 10.1111/j.1540-8167.1998.tb00096.x 9835268

[107] Kantoch MJ Qurashi MM Bulbul ZR Gorgels AP A newborn with a complex congenital heart disease, atrioventricular block, and torsade de pointes ventricular tachycardia. Pacing Clin Electrophysiol (1998) 21:2664–7 10.1111/j.1540-8159.1998.tb00043.x 9894657

[108] El-Hazmi MA al-Swailem AR Warsy AS al-Swailem AM Sulaimani R al-Meshari AA Consanguinity among the Saudi Arabian population. J Med Genet (1995) 32:623–6 10.1136/jmg.32.8.623 7473654 PMC1051637

[109] El Mouzan MI Al Salloum AA Al Herbish AS Qurachi MM Al Omar AA Consanguinity and major genetic disorders in Saudi children: a community-based cross-sectional study. Ann Saudi Med (2008) 28:169–73 10.4103/0256-4947.51726 18500181 PMC6074430

[110] Medeiros-Domingo A Bhuiyan ZA Tester DJ Hofman N Bikker H van Tintelen JP The RYR2-encoded ryanodine receptor/calcium release channel in patients diagnosed previously with either catecholaminergic polymorphic ventricular tachycardia or genotype negative, exercise-induced long QT syndrome: a comprehensive open reading frame mutational analysis. J Am Coll Cardiol (2009) 54:2065–74 10.1016/j.jacc.2009.08.022 19926015 PMC2880864

[111] Al-Aama JY Al-Ghamdi S Bdier AY Wilde AA Bhuiyan ZA De novo mutation in the *KCNQ1* gene causal to Jervell and Lange-Nielsen Syndrome. Clin Genet (2013). 10.1111/cge.12300 24125535

[112] Splawski I Timothy KW Tateyama M Clancy CE Malhotra A Beggs AH Variant of SCN5A sodium channel implicated in risk of cardiac arrhythmia. Science (2002) 297:1333–6 10.1126/science.1073569 12193783

[113] Wilde AA Bezzina CR Genetics of cardiac arrhythmias. Heart (2005) 91:1352–8 16162633 10.1136/hrt.2004.046334 PMC1769155

